# A Systematic Review on Transplantation Studies of the Retinal Pigment Epithelium in Animal Models

**DOI:** 10.3390/ijms21082719

**Published:** 2020-04-14

**Authors:** Céline Koster, Kimberley E. Wever, Ellie L. Wagstaff, Koen T. van den Hurk, Carlijn R. Hooijmans, Arthur A. Bergen

**Affiliations:** 1 Department of Clinical Genetics, Amsterdam University Medical Centers (AUMC), location Academic Medical Center (AMC), University of Amsterdam (UvA), 1105 AZ Amsterdam, The Netherlands; c.koster@amsterdamumc.nl (C.K.); e.l.wagstaff@amsterdamumc.nl (E.L.W.); k.t.vandenk@amsterdamumc.nl (K.T.v.d.H.); 2 Systematic Review Center for Laboratory Animal Experimentation (SYRCLE), Department for Health Evidence, Radboud Institute for Health Sciences, Radboud University Medical Center, 6525 GA Nijmegen, The Netherlands; kim.wever@radboudumc.nl (K.E.W.); carlijn.hooijmans@radboudumc.nl (C.R.H.); 3 Department of Anesthesiology, Pain and Palliative Medicine, Radboud University Medical Center, 6525 GA Nijmegen, The Netherlands; 4 Department of Ophthalmology, AUMC, AMC, UvA, 1105 AZ Amsterdam, The Netherlands; 5 Department of Ophthalmogenetics, Netherlands Institute for Neuroscience (NIN-KNAW), 1105 BA Amsterdam, The Netherlands

**Keywords:** retinal degenerative diseases, retinal pigment epithelium (RPE), cell therapy, systematic review, meta-analysis, transplantation

## Abstract

The retinal pigment epithelium (RPE) and the adjacent light-sensitive photoreceptors form a single functional unit lining the back of the eye. Both cell layers are essential for normal vision. RPE degeneration is usually followed by photoreceptor degeneration and vice versa. There are currently almost no effective therapies available for RPE disorders such as Stargardt disease, specific types of retinitis pigmentosa, and age-related macular degeneration. RPE replacement for these disorders, especially in later stages of the disease, may be one of the most promising future therapies. There is, however, no consensus regarding the optimal RPE source, delivery strategy, or the optimal experimental host in which to test RPE replacement therapy. Multiple RPE sources, delivery methods, and recipient animal models have been investigated, with variable results. So far, a systematic evaluation of the (variables influencing) efficacy of experimental RPE replacement parameters is lacking. Here we investigate the effect of RPE transplantation on vision and vision-based behavior in animal models of retinal degenerated diseases. In addition, we aim to explore the effect of RPE source used for transplantation, the method of intervention, and the animal model which is used. Methods: In this study, we systematically identified all publications concerning transplantation of RPE in experimental animal models targeting the improvement of vision (e.g., outcome measurements related to the morphology or function of the eye). A variety of characteristics, such as species, gender, and age of the animals but also cell type, number of cells, and other intervention characteristics were extracted from all studies. A risk of bias analysis was performed as well. Subsequently, all references describing one of the following outcomes were analyzed in depth in this systematic review: a-, b-, and c-wave amplitudes, vision-based, thickness analyses based on optical coherence tomography (OCT) data, and transplant survival based on scanning laser ophthalmoscopy (SLO) data. Meta-analyses were performed on the a- and b-wave amplitudes from electroretinography (ERG) data as well as data from vision-based behavioral assays. Results: original research articles met the inclusion criteria after two screening rounds. Overall, most studies were categorized as unclear regarding the risk of bias, because many experimental details were poorly reported. Twenty-three studies reporting one or more of the outcome measures of interest were eligible for either descriptive (thickness analyses based on OCT data; *n* = 2) or meta-analyses. RPE transplantation significantly increased ERG a-wave (Hedges’ g 1.181 (0.471–1.892), *n* = 6) and b-wave (Hedges’ g 1.734 (1.295–2.172), *n* = 42) amplitudes and improved vision-based behavior (Hedges’ g 1.018 (0.826–1.209), *n* = 96). Subgroup analyses revealed a significantly increased effect of the use of young and adolescent animals compared to adult animals. Moreover, transplanting more cells (in the range of 10^5^ versus in the range of 10^4^) resulted in a significantly increased effect on vision-based behavior as well. The origin of cells mattered as well. A significantly increased effect was found on vision-based behavior when using ARPE-19 and OpRegen^®^ RPE. Conclusions: This systematic review shows that RPE transplantation in animal models for retinal degeneration significantly increases a- and b- wave amplitudes and improves vision-related behavior. These effects appear to be more pronounced in young animals, when the number of transplanted cells is larger and when ARPE-19 and OpRegen^®^ RPE cells are used. We further emphasize that there is an urgent need for improving the reporting and methodological quality of animal experiments, to make such studies more comparable.

## 1. Introduction

The retinal pigment epithelium (RPE) is a neural epithelial cell layer lining the back of the eye [1]. The basal side of this polarized secretory cell layer rests on a collagen and elastin-rich extracellular matrix called the Bruch’s membrane (BM). Its apical side faces the photoreceptors. Together with the photoreceptors, the RPE is vital for normal retinal function and vision. The physiological roles of the RPE include defense against local oxidative stress, transport of nutrients and ions over the blood-retinal barrier, synthesis and excretion of growth factors, phagocytosis of the photoreceptor outer segments and the capture, storage and metabolism of vitamin A derivatives [1].

The importance of the RPE in vision is illustrated by its role in many retinal disorders. Patients of all age groups can be affected by diseases of the RPE. These disorders include usually rare, early-onset monogenic disorders such as albinism [2], fundus albipunctatus [3], some types of retinitis pigmentosa (RP) [4], Stargardt disease [5], gyrate atrophy [6], and bestrophinopathy [7].

However, the RPE is also involved in more common complex retinal diseases, such as age-related macular degeneration (AMD). In elderly patients, AMD affects 4% of the population over 60 and can be divided into dry and wet types. The majority of the patients suffer from dry or atrophic AMD which is characterized by the presence of drusen, pigment migration, and degeneration of the RPE and photoreceptors [8]. When dry AMD advances, the RPE degenerates almost completely and geographic atrophy characterized by RPE hypopigmentation and absence of the RPE sets in [9]. The wet types of AMD are characterized by choroidal neovascularization which causes bleedings, fluid accumulation and fibrosis within the macula [10]. Taken together, these RPE changes suggest that RPE dysfunction has a central role in AMD pathology and progression. Nonetheless, recent evidence suggests also that the photoreceptors, the choroid, as well as the blood contribute significantly to drusen formation and AMD disease pathology [11]. For the wet forms of AMD, a therapy exists which consists of monthly anti-VEGF injections. Monthly injections produce the best visual outcome, however intensive retreatment may not be sustainable in the long term. This treatment is based on hampering the disease progression however, a full cure is not available [12]. No effective treatment is currently available for the dry forms of AMD [8].

### RPE Replacements as Experimental Therapies in AMD

To date, no effective treatments for RPE degeneration in dry AMD exists. RPE replacement may be a promising future therapeutic option [13]. Previously, either a free graft of autologous RPE and choroid taken from the mid-periphery [14,15] or a suspension of RPE cells [16] were transplanted to the macular area of diseased retinas, with some long-term functional restoration in some patients treated with a sheet. However, autologous RPE cell suspensions which were harvested in the nasal area and placed in the macula area did not settle. Therefore, any successful RPE replacement therapy for AMD should also consider the need to (temporarily) replace the underlying BM [17]. It has been shown that the integrity of the BM decreases with age. Several changes occur in ageing BM: It thickens, may become calcified, and lipids accumulate within the BM [18]. This aged BM may not support the survival and differentiation of transplanted RPE into a polarized cell layer. The fate of transplanted cells depends on their ability to make new BM (components), which can replace the host’s BM. Temporarily restoring BM apart from the RPE would almost certainly be necessary, which implies further surgical manipulations beyond a simple subretinal injection of a cell suspension. 

Therapeutic RPE replacement strategies are currently tested in usually small clinical trials (phase I) for AMD and in some types of RP (China: NCT02755428, NCT03944239, NCT03046407; United States of America: NCT01345006, NCT01344993, NCT02286089). These strategies include cell suspension injection, transplantation of autologous RPE sheets, and transplantation of RPE-scaffolds to the subretinal space [19]. In parallel, preclinical trials are ongoing in both animal models with smaller eyes, such as mice and rats, or larger eyes, such as rabbits, pigs, and primates. Each type of model appears to have its own pros and cons. Mice and rats have the advantage that they can be genetically manipulated, that maintenance of strains is relatively cheap, and that they have short generation times. At the same time, their eye size and lack of morphological resemblance to the human eye seem to be the biggest limitations. In larger animal models, the advantages are bigger eye size and a similar morphology as the human eye. The limitations are the lack of (genetic) models and the costs. A few laser-, mechanically-, and chemically-induced large animal models are available but not often used so far [20,21]. Finally, in AMD, not only the RPE is affected but also the composition of BM is changing [22] and the choroid is thinning [23]. Thus, the ageing of the BM and choriocapillaris, in addition to the ageing RPE and retina, should also be considered when performing transplantation experiments in AMD animal models. Generally, the animals used in transplantation studies are young and have a healthy BM and healthy choriocapillaris [24]. This does not fully reflect the human situation.

In summary, the best strategy to test and improve the efficacy of RPE cell transplantations as a treatment for retinal degenerative diseases is currently not clear. In recent years many animal experimental studies have been published to illustrate the safety and (limited) effectiveness. However, little consensus about the approach and analyses exists. Regardless, human clinical (phase I) trials have started based on these variable results. So far, no systematic meta-analysis has been published to analyze the effects of RPE transplantation therapy in animal models for retinal degenerative diseases. Such a review can be beneficial for future experimental designs and provides a more solid basis for future clinical studies. Therefore, in this systematic review, we investigate the effect of RPE transplantation on vision and vision-based behavior in animal models of retinal degenerative disorders. We aim to explore the effect of RPE source used for transplantation, the method of intervention, and the animal models which were used.

## 2. Results

### 2.1. Search and Study Selection

**Figure 1:**
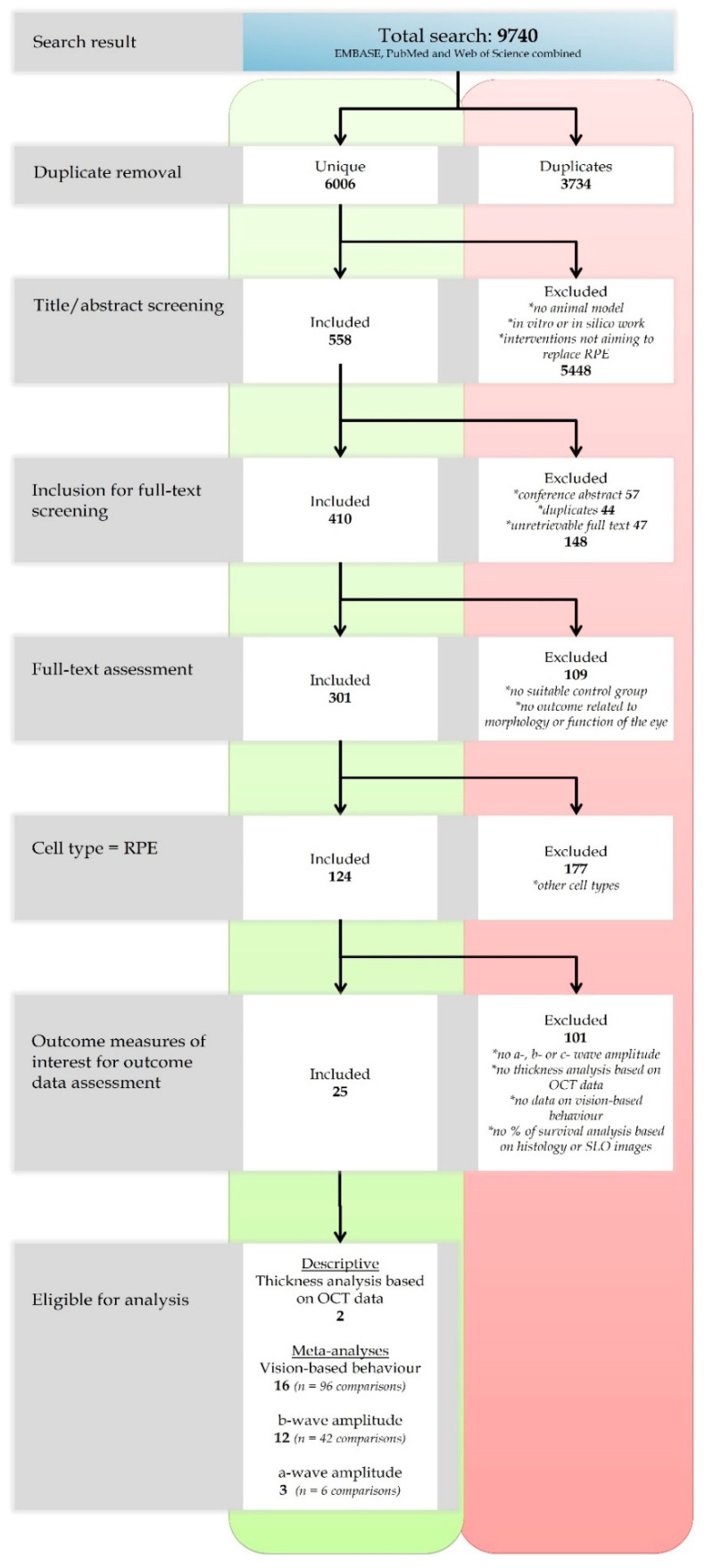
Flow diagram of the study selection progress. For details of the selection process: see text.

The study selection process is presented in Figure 1. We identified 9740 references. After removal of duplicates, we screened the titles and the abstracts of the remaining 6006 studies. After this first screening round, 5448 studies were excluded based on our predefined exclusion criteria. For the 558 remaining studies, we checked whether the full text was retrievable for full-text screening and whether those were original research articles. Three hundred and one studies were included for full-text screening. Finally, we selected the 124 studies that used transplanted RPE cells in experimental animal models targeting the improvement of vision (e.g., outcome measurements related to the morphology or function of the eye). Subsequently, all references describing one of the following outcomes were analyzed in depth in this systematic review (*n* = 25): a-, b-, and c-wave amplitudes, vision-based behavior, thickness analyses based on optical coherence tomography (OCT) data and transplant survival based on scanning laser ophthalmoscopy (SLO) data. Meta-analyses were performed on the a- (*n* = 3) and b-wave amplitudes (*n* = 12) from electroretinography (ERG) data as well as data from vision-based behavioral assays (*n* = 16). Retina thickness analyses based on OCT data (*n* = 2) was analyzed by descriptive synthesis.

### 2.2. Characteristics of the Included Studies

**Figure 2:**
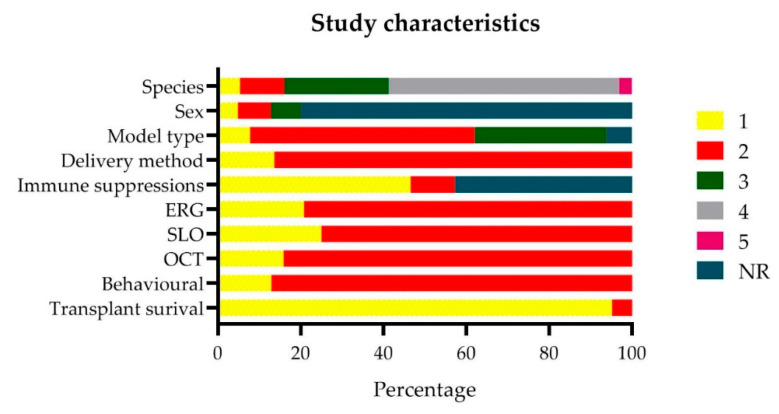
General study characteristics were extracted from the studies which were included in this systematic review. Species: 1 pig, 2 mouse, 3 rabbit, 4 rat, 5 other (cat, dog, *Macaca mulatta*, *Macaca fascicularis*). Sex: 1 female, 2 male, 3 both. Model type: 1 induced, 2 genetic, 3 not-applicable. Delivery method: 1 sheet, 2 suspension. Immune suppressions, ERG, SLO, OCT, behavioral and transplant survival: 1 yes, 2 no. NR = not reported.

The characteristics of the 124 included studies are summarized in Tables S1 and S2, and Figure 2. In general, there was a large variation in study characteristics. Most studies were performed in rats (56%). Other species included rabbit (25%), mouse (11%), pig (5%), and other species (cat, dog, *Macaca mulatta* and *Macaca fascicularis*, all 1%).

In the majority of studies (80%), the sex of animals used was not reported. When reported, roughly an equal number of males-only (8%), females-only (5%), and both sexes were used (7%). Most studies were performed in genetic animal models, e.g., rats or mice (54%). Chemically induced models were used in 8% of all cases.

Intervention methods included cells that were delivered in the subretinal space (100%) as a suspension (86%) or as a sheet (14%) in rats, rabbits, pigs, cats, and *Macaca mulatta*. Immune suppression was used in at least half of all studies (47%). Notably, 42% did not report whether any suppression was used.

All studies reported at least one of the following techniques to assess the outcome measures of interest: scanning laser ophthalmoscopy (SLO) was performed in 25% of the studies; electroretinography (ERG) in 20%; optical coherence tomography (OCT) in 16%; a behavioral assay in 13%; and, finally, transplant survival was determined in almost all studies (95%).

One outcome measure (retina thickness analysis based on OCT data) was selected for descriptive analysis. Out of 40 studies reporting any OCT data, only 2 performed a quantitative analysis on the retina thickness based on this data, which is not enough to perform a meta-analysis. Moreover, three outcome measures (a-wave amplitude, b-wave amplitude, and vision-based behavior) were selected for our meta-analysis since enough studies reported these quantitative data. Out of 26 studies reporting ERG data, only 12 were suitable for meta-analysis on the b-wave amplitude and only 3 were suitable for meta-analysis on the a-wave amplitudes. The remainder of 15 studies did report some ERG data, but they did not report any formal statistics on a-, b-, or c-wave amplitudes, thereby prohibiting meta-analysis on the reported data. All (16) studies which reported behavioral data were selected for meta-analysis because quantitative data and statistics were available. An overview of the key study characteristics of the 25 studies which were eligible for analysis is summarized in Table 1. A more extensive summary of the study characteristics is shown in Tables S1 and S2.

### 2.3. The Methodological Quality of the Studies

**Figure 3:**
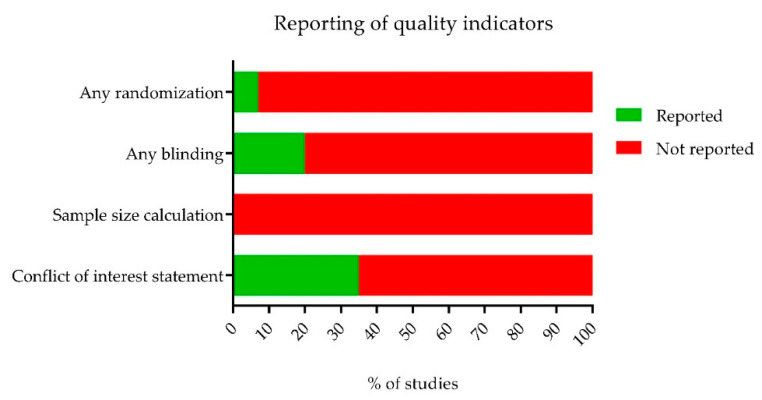
Reporting of four quality indicators in the 124 included studies. Only 7% of studies reported the use of any form of randomization, 20% reported the use of blinding and not one study reported a sample size calculation. In addition, only 35% of the studies reported a conflict of interest statement.

**Figure 4:**
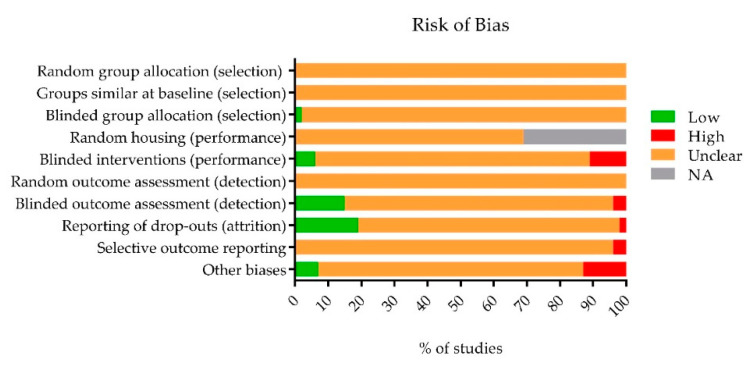
Using SYRCLE’s risk of bias tool [51], the risk of selection, performance, detection, attrition and other biases was assessed for the studies included in this review. The proportion of studies (%) which have a low, unclear or high risk of bias in several categories. As a consequence of poor reporting of measures to reduce several types of bias, the analysis resulted in a high percentage of “unclear” risk of bias for most items. NA (not applicable) holds true for studies that used an internal control in their experiments (e.g. one eye experimental group, other eye control group). Random housing between the experimental and control group is in that case, obviously, impossible.

Blinding and randomization of the experimental setup are essential measures to take to prevent statistical bias and underestimation or overestimation of the experimental data. However, both measures are infrequently reported in animal studies. We used SYRCLE’s risk of bias tool [50] to determine the risk of bias in all included studies. This risk of bias assessment is, in summary, presented in Figure 3 and Figure 4. We first assessed the reporting of four key study quality indicators, namely reporting of any randomization, any blinding, a sample size calculation and a conflict of interest statement. Only 7% of all included studies reported the use of any form of randomization, 20% reported the use of any blinding and not one study reported a sample size calculation. In addition, only 35% of the studies reported a conflict of interest statement. Although some authors mentioned applying randomization or blinding in their experiments, few adequately specified the methodology used. As a result, the majority of studies were assessed to have an unclear risk of most types of bias (Figure 4).

### 2.4. Descriptive Analysis 

#### Retina Thickness Analysis Based on OCT Data

Twenty of the included studies reported OCT data. Only two of them showed a quantitative analysis of the thickness of the retina (or a retinal cell layer). Both studies showed an increased effect on the thickness of the ONL in the group receiving an RPE transplantation [35,41].

### 2.5. Meta-Analyses

#### A-wave and B-wave Amplitudes Extracted from ERG Data

Twenty-six studies reported ERG data. Fourteen [46,52,53,54,55,56,57,58,59,60,61,62,63,64] of them were excluded from meta-analysis since they did not report quantitative data or only showed single representative ERG traces.

**Figure 5:**
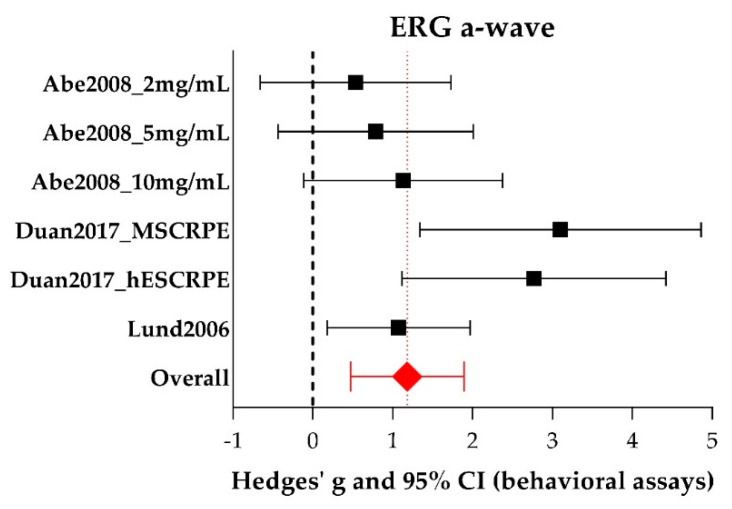
The meta-analysis of the effect of RPE transplantation on the size of the ERG a-wave amplitude. For every experimental group, Hedges’ g (a measure for effect size using smaller sample sizes, black solid squares) and its 95% confidence interval (CI) are graphically shown. The left side of the dashed black line (Hedges’ g = 0) favors the control group (untreated or sham-operated) and the right side favors the experimental group (RPE transplantation). RPE transplantation significantly increases the a-wave amplitude (Hedges’ g 1.181(0.471–1.892), red diamond).

Three studies [25,29,39] reporting six experiments were included in the meta-analysis of the ERG a-wave amplitude, all of them using rats. See Supplementary 1 for all study characteristics of the individual studies. The forest plot, in which the graphical representation of the meta-analysis is presented, is shown in Figure 5. Additional data (individual Hedges’ g (a measure for effect size using smaller sample sizes), lower- and upper limits) are shown in Table S3. For the a-wave meta-analysis, 45 experimental eyes and 22 control eyes were included. Group sizes were 5–14 (median = 5) for the experimental eyes and 3–8 (median = 8) for the control eyes.

Overall, RPE transplantation significantly increases a-wave amplitude (Hedges’ g 1.181 (0.471–1.892), *n* = 6). Overall, between-study heterogeneity was low (I^2^ = 26%), but this could be partly due to the limited number of studies in this meta-analysis. Subgroup analysis was not conducted for a-wave amplitudes because the subgroups contained too few studies in order to draw meaningful conclusions (less than 4 studies per subgroup remained).

**Figure 6:**
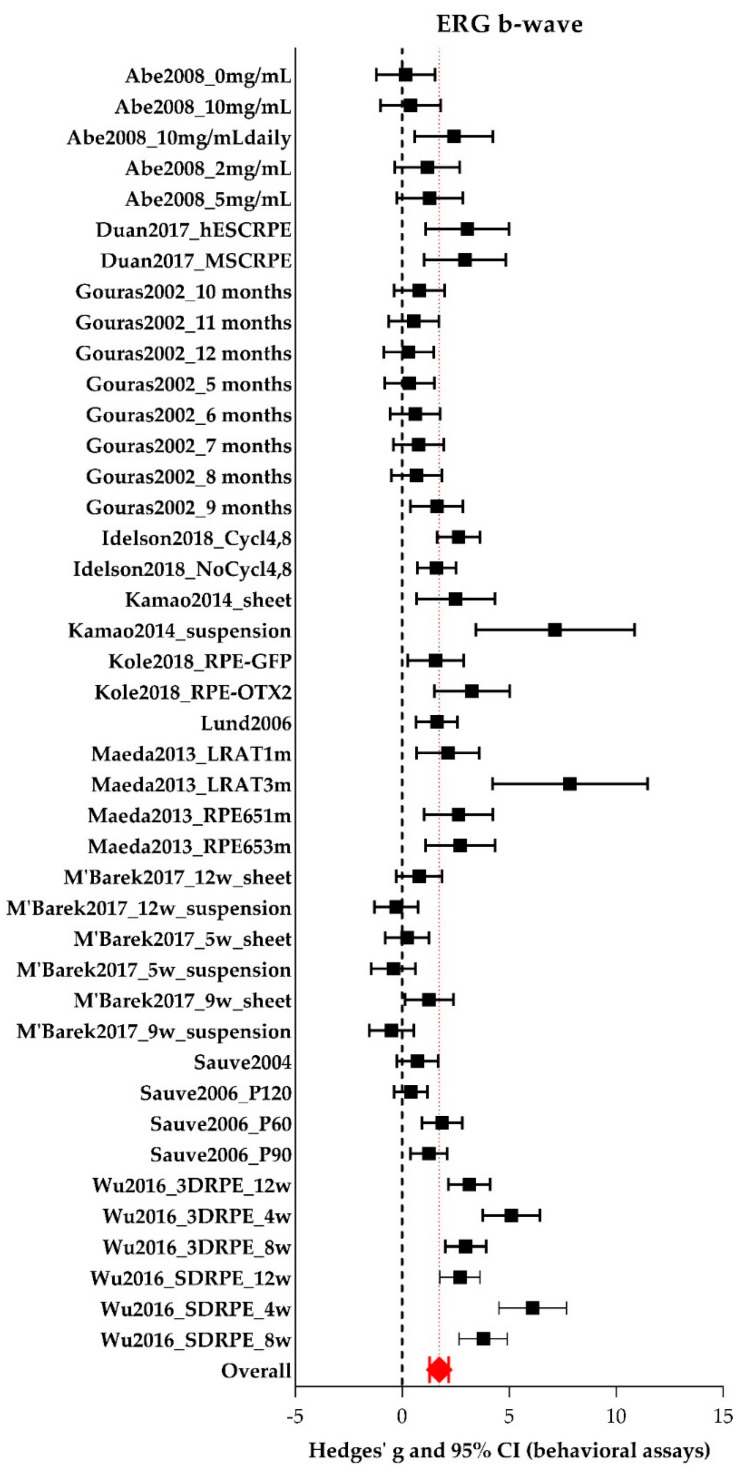
The meta-analysis on the effect of RPE transplantation on the ERG b-wave amplitude. For every experimental group, Hedges’ g (a measure for effect size using smaller sample sizes, black solid squares) and its 95% confidence interval (CI) are graphically shown. The left side of the dashed black line (Hedges’ g = 0) favors the control group (untreated or sham-operated) and the right side favors the experimental group (RPE transplantation). Overall, an RPE transplantation increased the b-wave amplitude significantly (Hedges’ g 1.734 (1.295–2.172), red diamond).

For the meta-analysis of ERG b-wave amplitude, twelve [25,29,30,32,33,35,39,40,41,44,45,49] studies reporting 42 experiments were included, of which 30 experiments were performed in rats and 12 in mice. See Supplementary 1 for all study characteristics of the individual studies. Four hundred and thirty-nine experimental eyes and 146 control eyes were included. The forest plot is shown in Figure 6. Additional data (individual Hedges’ g, lower- and upper limits) are presented in Table S4. Group sizes were 3–25 (median = 9) for the experimental eyes and 3–14 (median = 8) for the control eyes. Overall, RPE transplantation significantly increases b-wave amplitude (Hedges’ g 1.734 (1.295–2.172), *n* = 42). Between study heterogeneity was high (I^2^ = 82%). Subgroup analyses were performed and described below.

**Figure 7:**
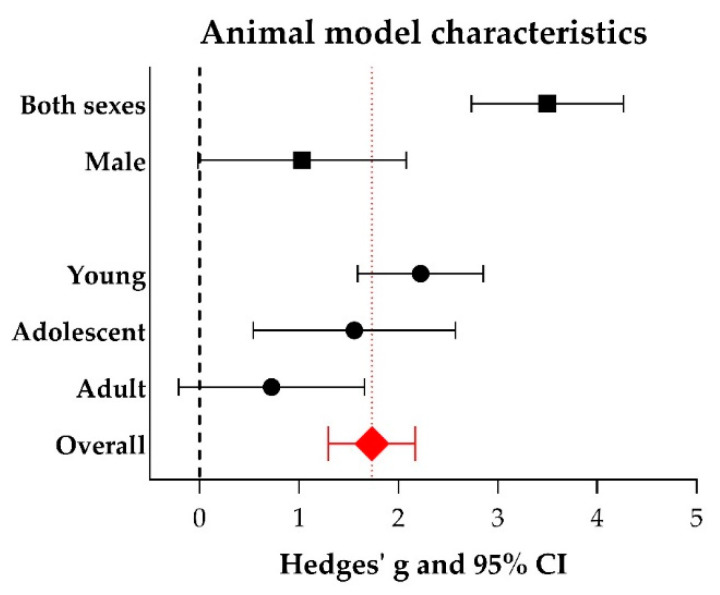
The effect of some animal model characteristics on the ERG b-wave amplitude. For every subgroup, Hedges’ g (a measure for effect size using smaller sample sizes, black solid squares) and its 95% confidence interval (CI) are graphically shown. The left side of the dashed black line (Hedges’ g = 0) favors the control group (untreated or sham-operated) and the right side favors the experimental group (RPE transplantation). An overall beneficial effect of RPE transplantation on the b-wave amplitude was observed (Hedges’ g 1.734 (1.295–2.172), red diamond). Significantly higher b-wave amplitudes were obtained when both sexes were used in the studies as compared to when males only were used (adjusted *p*-value 0.001). Significantly higher b-wave amplitudes were observed in young animals compared to adult animals (adjusted *p*-value 0.047).

The use of both sexes increases the b-wave amplitude significantly (Hedges’ g 3.500 (2.735–4.265), *n* = 8) compared to the use of males only (Hedges’ g 1.029 (−0.019–2.077), *n* = 5, adjusted *p*-value 0.001), see Figure 7. No studies within this meta-analysis reported using females only. Additional data (number of experiments, I^2^, Hedges’ g, its lower- and upper limits) are shown in Table S5. We further identified that the effect on b-wave amplitude was larger in young animals (< 20 days of age; Hedges’ g 2.223 (1.591–2.855), *n* = 18) compared to adult animals (> 2 months of age; Hedges’ g 0.724 (−0.210–1.658), *n* = 8, adjusted *p*-value 0.047). No significant difference could be observed between young and adolescent animals (20 days–2 months of age) or between adolescent and adult animals. Subgroup analyses were also performed for species (mouse versus rat), model type (genetic versus induced), genotype (*Mertk^−/−^* versus *Rpe65^−/−^* versus wildtype), number of cells which was transplanted (in the range of 10^3^ versus 10^4^ versus 10^5^), the cell type which was transplanted (ARPE-19 versus hESC-RPE versus pRPE versus BDNF-RPE), and the time after the intervention when the animals were analyzed (short-term (< 30 days) versus middle-term (30–90 days) versus long-term (> 90 days)). However, no significant differences were found. For other study characteristics, subgroups were too small (< 4 studies) to perform subgroup analysis.

### 2.6. Behavioral Assays

**Figure 8:**
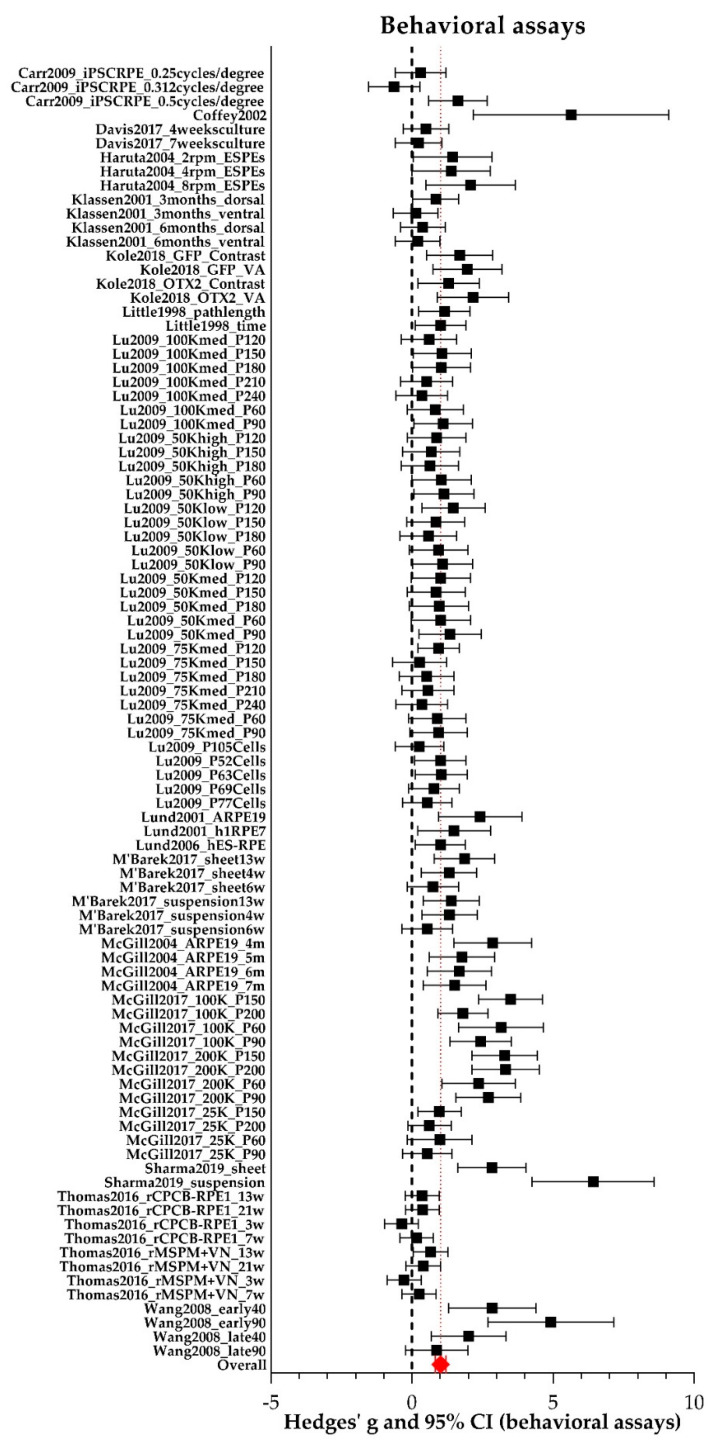
The meta-analysis on the effect of RPE transplantation on the outcome of vision-based behavioral assays. For every experimental group, Hedges’ g (a measure for effect size using smaller sample sizes, black solid squares) and its 95% confidence interval (CI) are graphically shown. The left side of the dashed black line (Hedges’ g = 0) favors the control group (untreated or sham-operated) and the right side favors the experimental group (RPE transplantation). Overall, RPE transplantation increased vision-based behavior (Hedges’ g 1.018 (0.826–1.209), red diamond).

Results for the outcome measure vision-based behavior are summarized in Figure 8. and additional statistics data (individual Hedges’ g, lower- and upper limits) can be found in Table S6. Sixteen studies [26,27,28,31,34,35,36,37,38,39,41,42,43,46,47,48] reporting 96 experiments were included in the meta-analysis. Ninety-one studies were performed in rats and five in mice. For all individual study characteristics see Supplementary 1. Group sizes were 4–59 (median = 8) and 2–21 (median = 7) for experimental eyes and control eyes respectively. In total, data of 1216 experimental eyes and 725 control eyes were available. Overall, between study heterogeneity was high (I^2^ = 73%). We observed significantly increased vision-based behavior in RPE transplanted eyes versus control eyes (Hedges’ g 1.018 (0.826–1.209), *n* = 96).

**Figure 9:**
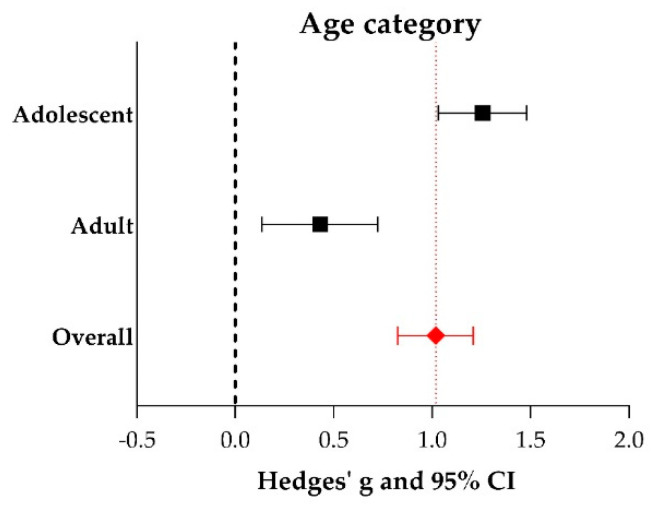
The effect of age on the outcome of vision-based behavioral assays. For every subgroup, Hedges’ g (a measure for effect size using smaller sample sizes, black solid squares) and its 95% confidence interval (CI) are graphically shown. The left side of the dashed black line (Hedges’ g = 0) favors the control group (untreated or sham-operated) and the right side favors the experimental group (RPE transplantation). Overall, RPE transplantation increased vision-based behavior (Hedges’ g 1.018 (0.826–1.209), red diamond). Significant better results were obtained when adolescent animals were used over adult animals (adjusted *p*-value 0.000033).

Subgroup analysis revealed that the use of adolescent animals (20 days–2 months of age; Hedges’g 1.257 (1.032–1.481), *n* = 59) increased the outcome of vision-based behavior significantly compared to the use of adult animals (> 2 months of age; Hedges’ g 0.431 (0.136–0.725), *n* = 31, adjusted *p*-value 0.000033), see Figure 9. No studies included in the meta-analysis of vision-based behavior reported the use of young animals. Additional data (number of experiments, I^2^, Hedges’ g, its lower- and upper limits) are shown in Table S7.

**Figure 10:**
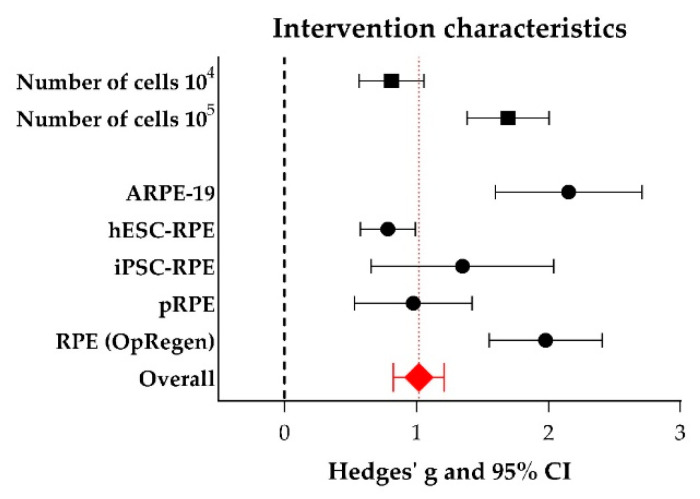
The effect of the number of transplanted cells and the origin of the cells on the outcome of vision-based behavioral assays. For every subgroup, Hedges’ g (a measure for effect size using smaller sample sizes, black solid squares) and its 95% confidence interval (CI) are graphically shown. The left side of the dashed black line (Hedges’ g = 0) favors the control group (untreated or sham-operated) and the right side favors the experimental group (RPE transplantation). Overall, RPE transplantation increased vision-based behavior (Hedges’ g 1.018 (0.826–1.209), red diamond). Significant better results were obtained when 10^5^ cells were transplanted when compared to 10^4^ cells (adjusted *p*-value 0.000036). The origin of the RPE cells which are transplanted matters as well, with ARPE-19 cells and OpRegen^®^ RPE being the best options.

In addition, subgroup analysis (Figure 10) showed that transplanting a larger number of RPE cells in the range of 10^5^ (Hedges’ g 1.694 (1.384–2.003), *n* = 32) yielded significant better vision-based behavior compared to the range of 10^4^ cells (Hedges’ g 0.811 (0.566–1.055), *n* = 47, adjusted *p*-value 0.000036). Furthermore, the origin of the RPE cells which were transplanted matters as well. The ARPE-19 cell line (Hedges’ g 2.153 (1.597–2.709), *n* = 10) resulted in significant increased vision-based behavior compared to hESC-RPE cells (Hedges’ g 0.783 (0.574–0.992), adjusted *p*-value 0.00035, *n* = 45). Interestingly, OpRegen^®^-RPE (Lineage Cell Therapeutics), which is currently in a clinical trial phase I (NCT02286089) (Hedges’ g 1.978 (1.551–2.406), *n* = 12), also resulted significantly in better vision-based behavior compared to hESC-RPE cells (adjusted *p*-value 0.000081) and primary RPE (Hedges’ g 0.976 (0.529–1.423), *n* = 10, adjusted *p*-value 0.048). Additional data (number of experiments, I^2^, Hedges’ g, its lower- and upper limits) are shown in Table S7. Subgroup analyses were also performed for species (mouse versus rat), genotype *(Mertk*^−/−^ versus *Elovl4*^−/−^), the delivery method (suspension versus sheet) and the time after the intervention when the animals were screened (short-term (< 30 days) versus middle-term (30–90 days) versus long-term (> 90 days). However, no significant differences between subgroups were found. For the other study characteristics (sex and immune suppressions), subgroups were too small (< 4 studies) to perform subgroup analysis.

### 2.7. Publication Bias

**Figure 11:**
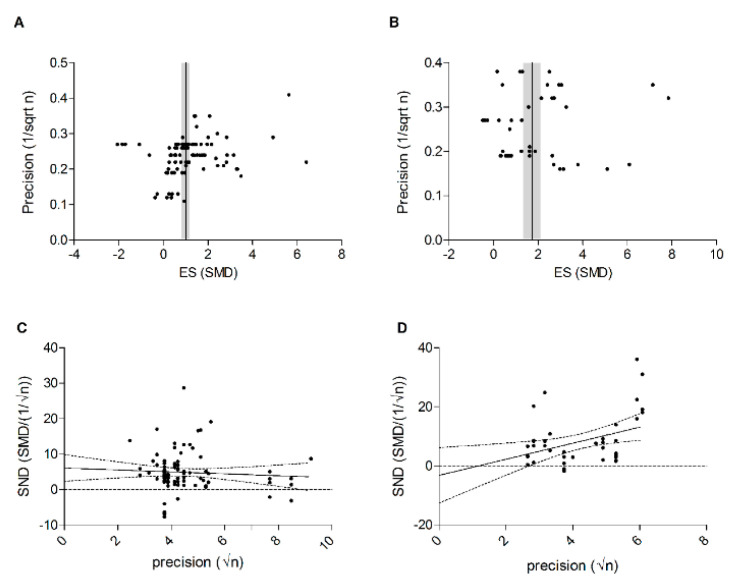
The funnel plots of studies which were included in the meta-analysis of (**A**) vision-based behavioral assays and (**B**) the b-wave amplitude. Publication bias was also assessed by Egger’s regression test, which is based on plotting the standardized normalized difference (SND) on the y-axis and the precision (the square-root of the number of animals in an experimental group) on the x-axis in a funnel plot (**C**,**D**). Indication for publication bias was found for the vision-based behavioral studies (Egger’s test, *p*-value = 0.003) and no indication for publication bias was found for the b-wave amplitude (Egger’s test, *p*-value = 0.44).

Publication bias was assessed by visual inspection of the funnel plots shown in Figure 11, panel A (outcome vision-based behavioral assays) and B (ERG b-wave amplitude). Slight asymmetry can be observed for both outcomes. In addition, we performed Egger’s test for small study effects, which indicated that small study effects are present for the outcome vision-based behavioral assays (*p*= 0.003) (Figure 11C), but not for the outcome ERG b-wave amplitude (*p* = 0.44) (Figure 11D). Data were insufficient to perform a similar analysis for the a-wave amplitude.

## 3. Discussion

The first (autologous) RPE replacement studies in animal models were performed in the 1970s [65]. After primary failures, the transplantations of human RPE (hRPE) to the eyes of animal models showed some prevention of photoreceptor death, rescued visual function to some extent and had some protective effects. However, as a rule of the thumb, results were variable [62,66,67,68].

This systematic review and meta-analysis provide a quantitative summary of available preclinical in vivo evidence of the effect of RPE transplantations in animal models for retinal degeneration on the a-wave amplitude, the b-wave amplitude and vision-based behavioral assays. Our review shows a significant increase in these three outcomes after RPE transplantation. The high variation between studies can partially be explained by the age and sex of the animals which served as hosts, and additionally by the number of cells and origin of RPE which was transplanted into the subretinal space. The increase of the b-wave amplitude was significantly larger when both sexes were used, compared to males-only, and when young animals were used compared to adult animals. The effect on vision-based behavioral assays was significantly larger when adolescent animals were used compared to adult animals. Moreover, the number of cells affects the intervention effect as well. A significantly larger effect on vision-based behavioral assays was found when more cells (in the range of 10^5^ versus 10^4^) was used and the cell type mattered as well; ARPE-19 and OpRegen^®^ RPE seemed to be the better options. The other study characteristics which were selected for subgroup analysis upfront did not explain any proportions of heterogeneity significantly, or could not be analyzed due to insufficient data. It is also important to consider the fact that some of the subgroup effects differed between the two outcomes. We did find a general effect of age of the animal. In both meta-analyses, a significantly larger effect was found for younger animals (young and adolescent) compared to adult animals. The other individual significant subgroup effects were not found in both meta-analyses. This could probably partly be explained by the fact of insufficient reporting of study characteristics. Moreover, insufficient reporting of study characteristics and the fact that most studies had an “unclear” risk of bias kept us from performing multiple subgroup analyses which might have explained some of the unexplained heterogeneity.

### 3.1. Methodological Limitations

Complete reporting of methodological details is essential to assess the risk of bias in original research articles and to determine the quality of the data. Reproducibility is very important in animal studies and variable results may be explained by differences in the methodology. However, insufficient reporting of methodology still occurs in the whole field of preclinical in vivo studies [65,66]. The risk of bias analysis within this systematic review revealed that details of most-importance regarding the study design are poorly reported, resulting in an “unclear” risk of bias for most (if not all) studies. Consequently, studies might have under- or overestimated their data which affects our meta-analysis [67]. Moreover, translational value to humans might be low because preclinical in vivo studies are generally not using sufficient animal numbers due to high costs [68,69]. Nonetheless, underpowered studies are included in this systematic review as well, because we wanted to give a full overview of the preclinical RPE transplantation field. Since none of the studies reported a power calculation or study protocol, the possibility cannot be excluded that the here presented meta-analyses suffer from an effect of such underpowering. Analyses revealed moderate to severe levels of heterogeneity between studies. We used a random-effects model to account for this heterogeneity and used subgroup analyses to explore the causes. Exploring this heterogeneity is one of the added values of meta-analyses of animal studies and might help to inform the design of future animal studies and subsequent clinical trials. Some of our most important findings in this paper, the importance of animal age, the transplanted cell number and origin of RPE cells which are transplanted, are a consequence of the information we obtained from exploring the sources of heterogeneity. Some risk of publication bias was found for the vision-based assays. As a result, neutral and negative results could be underrepresented, and the translational value might be mediocre.

### 3.2. Delivery Methods: Cell Suspensions or Monolayers?

According to our meta-analysis, the delivery method for RPE cells is not straightforward. Frequently, cells are delivered by means of an injection into the eye, mostly in the subretinal space. Intravitreal injections are hardly used since the transplanted RPE cells have to pass the retinal layers to reach the host’s RPE layer. Most recently it was postulated that RPE transplantation by means of a cell suspension injection might not be the most optimal procedure [69]. Dissociated cells that are injected into the subretinal space need to integrate into the host’s RPE layer and form a strong and new functional epithelial layer. This might be too much to ask of these cells. Therefore, transplantation of a sheet of cells might be a better strategy [70]. This is a sheet of cells only or it is a combination of a carrier (artificial BM) with a monolayer of RPE cells. However, the use of an RPE monolayer comes with yet another complication: Where an injection of a cell suspension is relatively simple, transplantation of a cell sheet (with or without a carrier) requires much more complex ocular surgery. Our meta-analyses revealed no significant difference of the effect of an RPE sheet versus an RPE suspension transplantation for both the b-wave amplitude (sheet *n* = 4 experimental groups, *n* = 3 studies; suspension *n* = 38 experimental groups, *n* = 12 studies) as the vision-based behavioral assays (sheet *n* = 11 experimental groups, *n* = 3 studies; suspension *n* = 85 experimental groups, *n* = 15 studies). However, we believe that we should take the limited amount of sheet transplantations compared to suspension transplantation into account when concluding anything on this matter.

### 3.3. Study Variables and Characteristics

Many factors contribute to cell survival following transplantation and improvement of the visual acuity of the patient. Normal RPE function is crucial for the maintenance of the outer blood-retinal barrier. In healthy eyes, the blood-retinal barrier provides an immune privilege and local tolerance to donor tissues. Nevertheless, non-matched cells are (eventually) rejected from the subretinal space over time unless systemic immune suppression is used [71].

Besides the delivery method, cell type, interventional timing, and site of graft placement are variables that could influence the outcomes of the trials. The clinical trials for cell-based therapies are in its infancy, so the extent of efficiency is unknown in humans. The main focus of these therapies is on the safety of the patients [19]. It is clear that current cell delivery systems need to be further improved and standardized.

### 3.4. RPE Cell Source and Immune Response

One important issue in transplantation is potential graft rejection. In summary, several different RPE cell sources have been used for transplantation purposes in animal models with variable success rates. These sources include autologous RPE, exogenic RPE, human donor RPE, cell lines such as ARPE-19 and RPE-J, stem cell-derived RPE (embryonic, induced pluripotent, adult), and progenitor cells. The first exogenous RPE transplantation study in animal models was performed by Li and Turner in 1988. They showed that healthy RPE cells from Long Evans rats can be grafted and can survive in the exogenous diseased retinal environment from the Royal College of Surgeons (RCS) rat [72,73]. However, human fetal RPE (hfRPE) cells transplanted into the eyes of rabbits resulted in inflammatory responses and rejected cells [74]. In more recent years, RPE cell lines, such as ARPE-19, and RPE cells, which were differentiated from stem cells, have been used for transplantation studies. These RPE lines yield, in principle, an unlimited transplantation source.

Our meta- and sub-group analysis showed that the cell type which is transplanted matters. The use of ARPE-19 and OpRegen^®^ RPE cells resulted in a significantly increased effect compared to hESC-RPE and iPSC-RPE when looking at the outcomes of the vision-based behavioral assays. We should note here that ARPE-19 and OpRegen^®^ RPE cells are a single cell type RPE source, whereas hESC-RPE and iPSC-RPE consist of more variable cell groups originating from multiple differentiation strategies [17,75,76]. Many recent papers state the advances in RPE differentiation protocols, however, there are still sources of variability. These sources include (stem) cell line, genetic background, passaging method, passage number, seeding density, and extracellular matrix [77,78]. This variability might explain the differences we find in our analysis between single cell types (ARPE-19 and OpRegen^®^) and more heterogeneous cell groups (hESC-RPE and iPSC-RPE).

Embryonic stem cell (ESC)-RPE cells or HLA-typed iPSCs can act as universal donors for large (sub-) populations since these cells are generally less immunogenic [79]. A number of safety studies showed that hESC-RPE cells could initiate an immune response in an AMD eye, possibly as an effect of the surgery or due to the diseased morphology [80]. Taken together, these data suggest that, despite the reputation of the eye as an immune-privileged site, non-autologous cells might be subject to graft rejection. In humans, personalized or HLA-typed iPSC-RPE cells could overcome this problem fully since they are an autologous cell source. In animal models, the use of low immune suppression regimes is essential for the success of the experiment.

In our meta-analysis, we found that roughly only half of the studies included reported the use of immune suppression. Only a few studies reported not to use them, and roughly half the other studies did not report anything on the use of immune suppression. We could not perform subgroup analysis on the use of immune suppressions, because too little studies reported the use. Therefore, we cannot make a clear statement on this topic. We would, therefore, recommend reporting whether or not immune suppression is used.

### 3.5. Small Eyes, Big Eyes

In the smaller eyed animal models, such as mice and rats, subretinal injections are performed using glass pipettes or small (e.g. 33/34 GA) needles. Since a large part of the vitreous cavity is filled with the lens, only a little space is left to place needles and other instruments. Another difficulty is the exact visualization of the procedure through the pupil of the small eye. Therefore, the transplantation of RPE sheets with or without carriers is still a challenge in animals with small eyes, which is hard, but especially in rats not impossible, to overcome.

In larger eyed animals, such as rabbits, pigs, and monkeys, the lens:vitreuscavity ratio is much smaller and more space is available to insert instruments. RPE sheets are usually placed using forceps or custom-made delivery tools [81,82,83]. Before placement of the graft, a complete vitrectomy is mostly performed, which is impossible in rodent eyes so far. Additionally, the transplantation site is enlarged by a subretinal injection using physiological salt. Vitreoretinal scissors are used to enable the implantation of the RPE sheet. Finally, multiple sclerotomies and retinotomies are required to perform a successful surgery. The surgery itself can be monitored using surgery microscopes with or without additional fundus visualization modules. In general, the studies in which functionality of the retina and vision was analyzed in a quantitative manner all used small animal models, such as mice and rats. Larger animal species, such as rabbits and pigs, may be used to better resemble the eye morphology of humans and the surgical procedures that come with it. Both small and large animal models have their pros and cons. Experimental costs rise extensively when using larger animal models. In the future, data of smaller and larger animal species might be separately re-analyzed and re-examined

### 3.6. Clinical Implications and Future Perspective

To our knowledge, this review is the first systematic overview of the effect of RPE transplantation in animal models for retinal degeneration. It provides useful insight into the methodology which is used worldwide in these preclinical in vivo studies. The unclear risk of bias is a reason to interpret the preclinical in vivo findings carefully. Overall, clinical trials are focusing on the safety and toxicology of RPE transplantations and consist still of too small groups to make clear statements about the efficacy (total number of enrolled patients 2–36, median = 12). Not all ongoing clinical trials are FDA approved (www.clinicaltrials.gov). We emphasize that there is an urgent need for improving the reporting and methodological quality of the conducted future animal experiments. Importantly, the effects of underpowering and publication bias should be avoided. Only on such a solid base, we can build designs of clinical trials which might be beneficial for patient groups.

## 4. Materials and Methods

This review is reported according to the PRISMA guidelines [84]. The inclusion criteria and analyses were specified in advance and documented in a protocol, registered at PROSPERO (https://www.crd.york.ac.uk/prospero/display_record.php?RecordID=79628) on 15 December 2017 (PROSPERO registration number CRD42017079628).

### 4.1. Adjustments to the Review Protocol

After study selection according to the protocol’s criteria, we found that the location of delivery correlated completely with the cell type which was transplanted. Therefore, and because of the large number of studies that were included, we decided to include all studies which transplanted RPE cells, which were all transplanted in the subretinal space, in this review. The other cell types will be analyzed in subsequent systematic reviews. We also conducted the following additional post hoc subgroup analyses (not listed in the original protocol): the effects of the number of cells that were transplanted, the follow-up time and whether or not immunosuppression was used.

### 4.2. Search Strategy

A comprehensive literature search was performed in PubMed, EMBASE (via OvidSP) and Web of Science to identify relevant articles until the 26^th^ of September 2019. The search strategy (see Supplementary 1) consisted of the component “retinal degenerative diseases” and “cell transplantation replacing existing RPE”. For PubMed and EMBASE, these were used in combination with a search filter for animal studies [51,85]. The search strategy was designed by information specialists at the Radboud University Medical Center (Nijmegen, the Netherlands) in collaboration with the medical library of the Amsterdam University Medical Centers (AUMC, location AMC, Amsterdam, The Netherlands).

### 4.3. Study Selection

After obtaining all references, duplicates were removed. Subsequently, unique references that were screened for relevance based on their title and abstract in Rayyan. A reference was included when it was an original full research article, using an animal model to study retinal degenerative diseases, included an appropriate control group receiving no treatment or a placebo/sham, it concerned a transplantation of cells replacing existing RPE, it reported outcome measurements related to the morphology or function of the eye. No language or publication date restrictions were made. In case of any doubt due to the absence of the abstract or insufficient information to make a valid judgment, references were included for full-text screening in Rayyan. Full-text copies of all publications eligible for inclusion were subsequently assessed and included when they met our pre-specified inclusion criteria. Whenever necessary, studies in a language other than English were translated using Google Translate. In case of any doubts, a native speaker was asked to extract the data necessary for this review. For both screening phases, references were screened in duplicate by two independent reviewers and in case of disagreement between the reviewers, a third reviewer served as arbiter.

### 4.4. Study Characteristics

Study characteristics were extracted from the studies. Bibliographical information (author, year of publication, journal), animal model characteristics (species, strain, sex, age, weight, disease, control type, genotype, induction method), experimental characteristics (donor species, donor strain, cell type, number of transplanted cells/surface, administration place, type of intervention, carrier medium, injection volume, scaffold type, immune suppressors) and outcome characteristics (ERG, SLO, OCT, behavioral, transplant survival) were obtained. For all studies included in a meta-analysis, group averages (mean, standard deviation (SD), standard error (SE) number of animals per group (*n*), and the number of eyes per group (*n*)) were extracted for all outcome measures. When the SE was reported, the SD was recalculated from it using the number of eyes investigated. If a study reported data from several experimental groups, it was extracted as separate comparisons. Attempts were made to obtain original data by contacting authors if results were presented incompletely. If there was no reply within two weeks after sending a reminder, the data was not included in the meta-analysis. Graphically presented data was extracted as numerical data using ImageJ (version 1.8.0, National Institutes of Health, Bethesda, MD, United States of America). 

### 4.5. Risk of Bias Assessment

The risk of bias was assessed for all studies which were included in the review after the full-text screening. The internal validity of the included studies was assessed in duplicate by two independent reviewers using SYRCLE’s risk of bias tool [50]. This tool contains ten entries which are related to six types of bias (selection, performance, detection, attrition, reporting and ‘other’). Disagreements between reviewers were resolved through discussion. For all studies, we determined whether the study used an external (other animal) or an internal (same animal, other eye) control group. For studies using an external control, all items of the risk of bias tool were scored. For studies using an internal control, all items except item 4 (random housing) were scored. Some studies reported that one specific eye was always used as the experimental eye. This causes a high risk of bias for some items (e.g., 5 and 7). The risk of bias was assessed with the eye, which received the transplant, itself as an experimental unit and not the animal. Publication bias was assessed by visual inspection of the funnel plots and conducting Egger’s regression test. Of note, since the effect measure used for both outcomes was Hedges’ g, we used 1 divided by the square root of the total number of animals (1/sqrt n) as our precision estimate in publication bias analyses [86].

### 4.6. Data Analysis

Meta-analysis was performed whenever at least four or more independent studies reported on a specific outcome measure. As a consequence, meta-analysis could only be performed for three of our outcomes (the a-wave amplitude, the b-wave amplitude and the outcome of vision-based behavioral assays) using Comprehensive Meta-Analysis Software (CMA). Vision-based behavioral assays included optokinetic reflex, pupillometry, and visual water tasks. The effect size was expressed as a standardized mean difference (SMD (Hedges g); the mean of the experimental group minus the mean of the control group divided by the pooled SDs of the two groups) and its 95% confidence interval (95% CI). To enable assessment of sources of anticipated between-study heterogeneity, the individual effect sizes were subsequently pooled to obtain an overall SMD and 95% CI. Because of the exploratory nature of animal studies, a random-effects model was used, which takes the anticipated between-study heterogeneity into account. Heterogeneity was addressed as I^2^, i.e., the proportion of the total observed heterogeneity that can be explained by between-study heterogeneity. Subgroup analyses were performed when subgroups contained a minimum of four independent comparisons. Subgroups were pre-specified in our protocol and analyses were planned for animal species, sex, age, intervention method (suspension/sheet), the cell type which was transplanted, type of animal model (genetic/induced), and genotype. In addition, some post hoc analyses were conducted for the number of cells, time after the intervention and whether immune suppressions were used (Y/N) as stated in the section adjustments made to the protocol. When a study contained multiple control groups, the control group undergoing the same surgical procedure as the experimental group (sham) was used. If multiple experimental groups were compared to the same control group, the number of animals in the control group was divided by the number of treatment groups, with a minimum of two animals in the control group and the number was rounded up. If the standard deviation was 0, the standard deviation of a similar group within that same reference was used in the meta-analysis. The *p*-value was adjusted for multiple testing, with an adjusted *p*-value of 0.05 and lower to be considered significant.

## 5. Conclusions

This systematic review shows that RPE transplantation in animal models for retinal degeneration significantly increases a- and b- wave amplitudes and improves vision-related behavior. These effects appear to be more pronounced in young animals, when the number of transplanted cells is larger and when ARPE-19 and OpRegen^®^ RPE cells are used.

Overall, this review clearly revealed that methodological details of animal experiments are often poorly reported. Although this is not unique to the ophthalmology field, it is worrying as a lack of reporting important methodological details will to some extent indicate neglected use of these methods to reduce bias, which can cause skewed results. This may seriously hamper drawing reliable conclusions from the included animal studies.

## Figures and Tables

**Table 1:** An overview of the study characteristics of the 25 studies which were eligible for in depth analysis. WT = wildtype; NA = not applicable; NR = not reported; NaIO_3_ = sodium iodate; i.v. = intravenous; pRPE = primary RPE; hESC = human embryonic stem cell; BMSC = bone marrow-derived mesenchymal stem cell; 3D = three dimensions; SD = single dimension; CPCB-VN = parylene C membrane with vitronectin; PLGA = poly lactic-co-glycolic acid; N = no; Y = yes.

Author Year	Species	Genotype	Induction Method	Donor Species	Cell Type	Suspension or Sheet	Injection Volume (µL)	Scaffold	Immune Suppression	ERG	SLO	OCT	Behavioral
**Abe2008** [25]	Rat	WT	Light	Rat	pRPE	Suspension	2	NA	N	Y	N	N	N
**Carr2009** [26]	Rat	MERTK^−/−^	NA	Human	pRPE	Suspension	2	NA	Y	N	N	N	Y
**Coffey2002** [27]	Rat	MERTK^−/−^	NA	Human	ARPE-19	Suspension	2	NA	Y	N	N	N	Y
**Davis2017** [28]	Rat	MERTK^−/−^	NA	Human	hESC-RPE	Suspension	1.5	NA	Y	N	N	N	Y
**Duan2017** [29]	Rat	WT	NaIO_3_; i.v.; 50 mg/kg	Human	hBMSC; BMSC-RPE; hESC-RPE	Suspension	2	NA	Y	y	N	N	N
**Gouras2002** [30]	Mouse	RPE65^−/−^	NA	Mouse	pRPE	Suspension	10	NA	NR	Y	N	N	N
**Haruta2004** [31]	Rat	MERTK^−/−^	NA	Monkey	hESC-RPE	Suspension	3	NA	Y	N	N	N	Y
**Idelson2018** [32]	Rat	MERTK^−/−^	NA	Human	hESC-RPE	Suspension	2–3	NA	Y	Y	Y	N	N
**Kamao2014** [33]	Rat; Macaca fascilaris	MERTK^−/−^; NR	NA; NR	Human; Monkey	pRPE	Suspension; Sheet	2; 100	none	Y; NR	Y; N	Y	N; Y	N
**Klassen2001** [34]	Rat	MERTK^−/−^; WT	NA	Rat	pRPE	Suspension	2	NA	NR	N	N	N	Y
**Kole2018** [35]	Rat	MERTK^−/−^	NA	Pig	pRPE	Suspension	2	NA	Y	Y	Y	Y	Y
**Little1998** [36]	Rat	MERTK^−/−^	NA	Human	pRPE	Sheet	3–5	NA	Y	N	N	N	Y
**Lu2009** [37]	Rat; Mouse	MERTK^−/−^; ELOVL4^−/−^	NA	Human	pRPE	Suspension	2	NA	Y	N	N	N	Y
**Lund2001** [38]	Rat	MERTK^−/−^	NA	Human	ARPE-19; h1RPE7	Suspension	2	NA	Y	N	N	N	Y
**Lund2006** [39]	Rat	MERTK^−/−^	NA	Human	pRPE	Suspension	NR	NA	Y	Y	N	N	Y
**Maeda2013** [40]	Mouse	LRAT^−/−^; RPE65^−/−^ Tyrc-2J/J	NA	Mouse; Human	pRPE; iPSC-RPE	Suspension	1.5	NA	Y	Y	Y	Y	N
**M’Barek2017** [41]	Rat	MERTK^−/−^	NA	Human	hESC-RPE	Sheet; Suspension	NA; 4	Gelatin; NA	Y	Y	N	Y	Y
**McGill2004** [42]	Rat	MERTK^−/−^	NA	Human	ARPE-19	Suspension	2	NA	Y	N	N	N	Y
**McGill2017** [43]	Rat	MERTK^−/−^	NA	Human	RPE (OpRegen)	Suspension	2	NA	Y	N	N	N	Y
**Sauve2004** [44]	Rat	MERTK^−/−^	NA	Human	ARPE-19	Suspension	2	NA	Y	Y	N	N	N
**Sauve2006** [45]	Rat	MERTK^−/−^	NA	Human	ARPE-19	Suspension	2	NA	Y	Y	N	N	N
**Sharma2019** [46]	Rat; Pig	MERTK^−/−^	Laser	Human	iPSC-RPE	Sheet; Suspension	2	PLGA	Y	Y	Y	Y	Y
**Thomas2016** [47]	Rat	MERTK^−/−^	NA	Human	pRPE	Sheet	NA	CPCB-VN	Y	N	N	N	Y
**Wang2008** [48]	Rat	MERTK^−/−^	NA	Human	ARPE-19	Suspension	3	NA	Y	N	N	N	Y
**Wu2016** [49]	Rat	MERTK^−/−^	NA	Human	3DRPE; SDRPE	Suspension	1–2	NA	Y	Y	N	N	N

## Supplementary Materials

Supplementary materials can be found at https://www.mdpi.com/1422-0067/21/8/2719/s1. Supplementary 1: Complete search strategy for PubMed, Web of Science and EMBASE. Supplementary 2: Study characteristics including all references. Supplementary 3: Statistics of meta-analyses and subgroup analyses including references.

## Abbreviations

3D	Three Dimensions
AMD	Age-related Macular Degeneration
ARRIVE	Animal Research: Reporting of In Vivo Experiments
BM	Bruch’s Membrane
BMSC	Bone Marrow-derived Mesenchymal Stem Cell
CI	Confidence Interval
CMA	Comprehensive Meta-analysis Software
CPCB	Parylene C Membrane
Cycl	Cyclosporine
ERG	Electroretinography
FDA	Food and Drug Administration
GA	Gauge
GFP	Green Fluorescent Protein
Hedges’ g	Measure of Effect Size
hESC	Human embryonic Stem Cell
hfRPE	Human Fetal Retinal Pigment Epithelium
HLA	Human Leukocyte Antigen
I^2^	Measure of Heterogeneity
iPSC	Induced Pluripotent Stem Cell
LRAT	Lecithin Retinol Acyltransferase
MERTK	Proto-oncogene Tyrosine-protein Kinase Mer
MSC	Mesenchymal Stem Cell
MSPM	Mesh-supported Parylene C Membrane
NA	Not Applicable
NR	Not Reported
OCT	Optical Coherence Tomography
ONL	Outer Nuclear Layer
OTX2	Orthodenticle Homeobox 2
PLGA	Poly Lactic-co-glycolic Acid
PROSPERO	International Prospective Register of Systematic Reviews
pRPE	Primary Retinal Pigment Epithelium
RPE	Retinal Pigment Epithelium
RPE65	Retinal Pigment Epithelium-specific Protein 65 Kilodalton
SD	Single Dimension
SD	Standard Deviation
SE	Standard Error
SLO	Scanning Laser Ophthalmoscopy
SMD	Standard Mean Difference
SYRCLE	Systematic Review Center for Laboratory Animal Experimentation
VN	Vitronectin
